# Hepatic Infarction in a Patient With Sickle Cell Trait Presenting With HELLP Syndrome

**DOI:** 10.7759/cureus.23724

**Published:** 2022-04-01

**Authors:** Anish C Paudel, John F Altomare, Oluwaseun Shogbesan, Andrew Lee, Parth Desai, Jesus S Noain, Suravi Khanal

**Affiliations:** 1 Internal Medicine, The Reading Hospital, West Reading, USA; 2 Gastroenterology, The Reading Hospital, West Reading, USA; 3 Internal Medicine, Manipal College of Medical Sciences, Pokhara, NPL

**Keywords:** elevated liver enzymes, preeclampsia, sickle cell trait, hellp syndrome, hepatic infarction

## Abstract

Hepatic infarction is uncommon due to the dual blood supply from the hepatic artery and portal vein. The majority of the cases are caused following liver transplant or hepatobiliary surgery, hepatic artery occlusion, or shock. Hepatic infarction is a rare complication of hemolysis, elevated liver enzymes, and low platelet (HELLP) syndrome. HELLP is an obstetrical emergency requiring prompt delivery. The presence of elevated liver enzymes, mainly alanine aminotransferase and aspartate aminotransferase in pre-eclampsia, should warrant diagnosis and treatment in the line of HELLP syndrome. Our patient with underlying sickle cell trait presented with features of HELLP syndrome in her third trimester of pregnancy. She underwent cesarean delivery on the same day of the presentation. The liver enzymes continued to rise following delivery and peaked on postoperative day two. Contrast computed tomography scan showed multifocal hepatic infarctions. Pre-eclampsia by itself is a state of impaired oxygenation and can lead to hepatic hypoperfusion, and appeared to be a clear contributor to the hepatic infarction in this case. However, this case also raises the question of whether the underlying sickle cell trait might have potentiated the hepatic infarction. Although sickle cell disease is well known to cause hepatic infarctions, it is unknown whether the sickle cell trait affects the liver to a similar extent as sickle cell disease. In addition, there have been case reports of sickle cell trait causing splenic infarcts and renal papillary necrosis, but it remains unclear if it can be directly associated with hepatic infarction.

## Introduction

Hemolysis, elevated liver enzymes, and low platelets (HELLP) syndrome is a feared complication of pre-eclampsia. Elevated liver enzymes indicate end-organ damage which mandates prompt management to prevent fetal and maternal mortality. Although hepatic infarction is rare because of the liver’s dual blood supply from the hepatic artery and portal vein, there have been cases described in the literature. Unlike sickle cell disease, sickle cell traits do not commonly cause life-threatening vaso-occlusive crises. Here, we present the case of a young patient with sickle cell trait who developed hepatic infarction in the setting of severe pre-eclampsia.

## Case presentation

A 19-year-old nulliparous patient with a medical history of sickle cell trait presented at 34 weeks gestation with two weeks of sharp, intermittent right upper quadrant (RUQ) pain and headache. Her blood pressure was 180/103 mmHg at presentation. Physical examination revealed RUQ tenderness, 2+ pitting edema over bilateral lower extremities, and a gravid uterus with fetal movements.

Initial labs showed elevated liver enzymes and decreased hemoglobin 11.3 (normal: 12-16) g/dL and platelet count 115 (normal: 130-400) 10^3^/µL. Spot urine total protein was >2,000 mg/dL (normal: <300 mg/dl in pregnant women). Total bilirubin levels and acute hepatitis panel were normal. Peripheral blood smear revealed +2 schistocytes and reduced platelets without clumping. RUQ ultrasound showed extensive hepatic steatosis without discrete hepatic mass, gallbladder thickening without choledocholithiasis, and no biliary dilatation.

**Figure 1:**
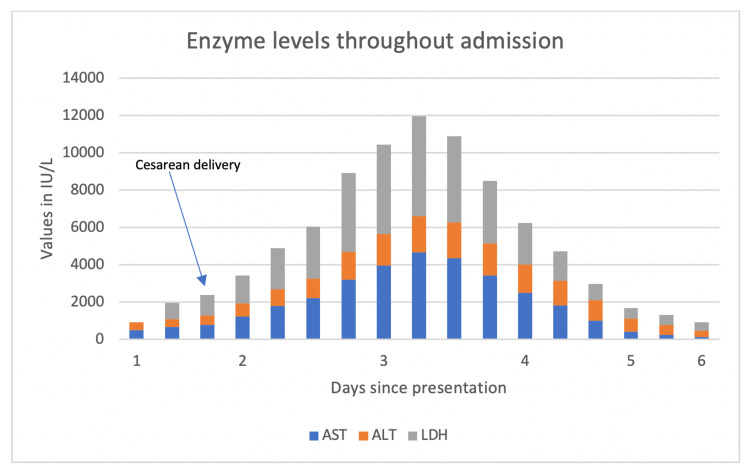
Enzyme levels during hospitalization. AST: aspartate aminotransferase; ALT: alanine aminotransferase; LDH: lactate dehydrogenase

She was diagnosed with severe pre-eclampsia with HELLP syndrome. The patient received intravenous labetalol and magnesium and underwent a successful cesarean section on the same day. The liver enzymes continued to rise and peaked on the third postoperative day (Figure 1).

Alkaline phosphatase peaked at 145 (normal: 34-104) IU/L, aspartate aminotransferase peaked from 487 to 4,680 (normal: 13-39) IU/L, alanine aminotransferase peaked from 426 to 1,925 (normal: 7-52) IU/L, and lactate dehydrogenase peaked from 867 to 5,362 (normal: 140-271) IU/L. Haptoglobin was <30. The platelets reached a nadir at 36 (normal: 130-400) 10^3^/µL, and hemoglobin reached a nadir at 8.0 (normal: 12-16) g/dL; by postoperative day three, they started to improve.

**Figure 2:**
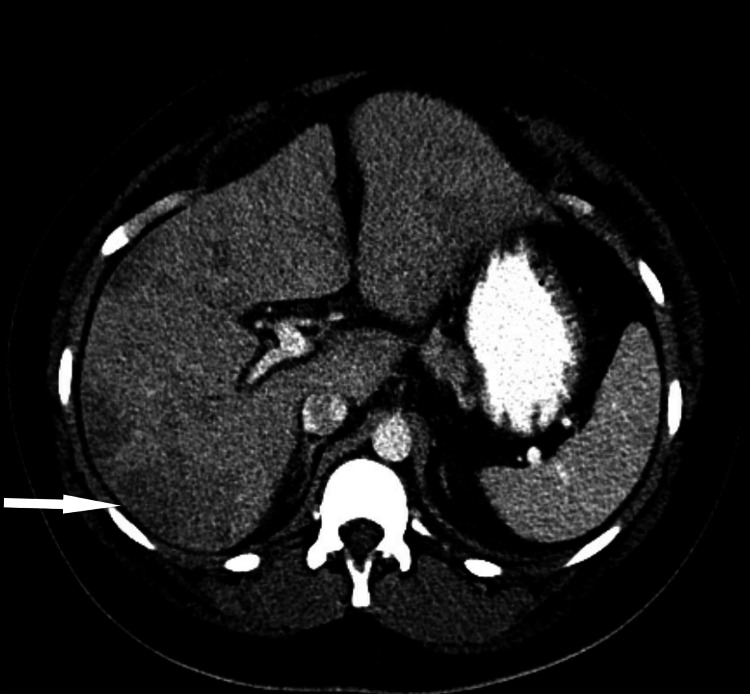
Contrast CT of the abdomen showing areas of hepatic infarction. CT: computed tomography

A contrast computed tomography scan (Figure 2) revealed multiple hepatic perfusion abnormalities suggestive of multifocal hepatic infarctions without subcapsular hematoma or hemorrhage. She was treated symptomatically and did not require blood transfusions, steroids, or intravenous immunoglobulins. The patient was discharged home four days later. Unfortunately, she was lost to follow-up.

## Discussion

HELLP syndrome indicates severe pre-eclampsia. Pre-eclampsia is diagnosed when a previously normotensive woman with a gestational age of >20 weeks has (1) systolic blood pressure (SBP) of ≥140 mmHg or diastolic blood pressure (DBP) of ≥90 mmHg measured on two different occasions at least four hours apart and (2) proteinuria of ≥300 mg/day [1]. Pre-eclampsia is considered to be severe with SBP of ≥160 mmHg and/or DBP of >110 mmHg and/or features of end-organ damage [2]. HELLP syndrome has been shown to complicate 10-20% of pregnancies associated with pre-eclampsia [3]. Hepatic infarction is often rare due to the dual blood supply of the liver [4]. Nonetheless, there have been cases described in the literature [5,6]. Subcapsular hematoma is a rare complication of HELLP syndrome and can rupture, leading to hemoperitoneum and shock [7,8].

The mainstay of treatment for HELLP syndrome associated with pre-eclampsia is prompt delivery [3]. Medical management is focused on supportive care of the mother, prevention or management of end-organ dysfunction, and the use of corticosteroids to allow for fetal lung maturation. In addition, intravenous magnesium sulfate is used to prevent seizures in such patients [9]. Plasmapheresis has also been used in the treatment of HELLP syndrome [10]. A recent literature review mentioned 36 patients who received liver transplants following HELLP syndrome. Liver failure and hemorrhagic shock were two of the most common indications for liver transplants [10]. Our patient, fortunately, did well with symptomatic blood pressure control and prompt delivery. Sickle cell disease is a severe illness with abnormal hemoglobin caused by two sickle (S) genes. Sickle cell trait patients, on the other hand, have a normal hemoglobin structure as they have one abnormal and one normal gene. Sickle cell trait has been associated with renal papillary necrosis and splenic infarctions, especially at high altitudes; however, little is known about its association with hepatic infarction [11,12]. Although sickle hepatopathy is a term used to describe patterns of liver dysfunction in patients with sickle cell disease, there has been no mention of sickle cell trait causing liver infarction just by itself in the literature [13].​​ Preeclampsia by itself is a disease resulting from abnormal vasculature within the uteroplacental system. The defective spiral artery remodeling leads to villous damage resulting in underlying vasoconstriction. Thus, the ongoing ischemia-reperfusion injury leads to impaired oxygenation and tissue hypoxia [14]. Our patient presented with features of HELLP syndrome, but it is not fully known whether the underlying sickle cell trait could have contributed to the infarction in the setting of tissue hypoxia. This case highlights the importance of prompt recognition and management of HELLP syndrome in pregnant patients with pre-eclampsia. Extra caution may be warranted when the patient has underlying hemoglobinopathies. Early coordination between hepatologists and obstetricians is vital given the high fetal and maternal mortality rate. Failure to do so can lead to complications, including hepatic failure, subcapsular hematoma, hepatic rupture, disseminated intravascular coagulation, and ultimately death.

## Conclusions

HELLP syndrome is a feared complication of preeclampsia, which in severe cases can lead to life-threatening hepatic infarction. Furthermore, extra caution is warranted in the setting of underlying hemoglobinopathies. Our patient additionally had an underlying sickle cell trait that has been shown to cause splenic infarcts and renal papillary necrosis. Sickle cell hepatopathy comprises a range of hepatic complications resulting from sickle cell disease. The complications can be secondary to the sickling of the hemoglobin itself, due to chronic hemolysis, or as a result of multiple blood transfusions. Sickle cell trait, on the other hand, is considered to be a milder form of the disease when a person has one defective allele of the beta hemoglobin gene instead of both defective alleles seen in patients with sickle cell disease. Hepatic involvement is rare in sickle cell traits. Our patient with sickle cell trait was found to have hepatic infarctions in the context of underlying preeclampsia, a disease causing impaired oxygenation between fetal-maternal circulation. We conclude that despite the dual blood supply to the liver, patients with sickle cell traits might also potentiate hepatic infarctions, especially in the presence of a concurrent hypoperfusion state.
